# Fukushima radionuclides in the NW Pacific, and assessment of doses for Japanese and world population from ingestion of seafood

**DOI:** 10.1038/srep09016

**Published:** 2015-03-12

**Authors:** Pavel P. Povinec, Katsumi Hirose

**Affiliations:** 1 Department of Nuclear Physics and Biophysics, Comenius University, Bratislava, Slovakia; 2 Department of Materials and Life Sciences, Sophia University, Tokyo, Japan

## Abstract

Variations of Fukushima-derived radionuclides (^90^Sr, ^134^Cs and ^137^Cs) in seawater and biota offshore Fukushima and in the NW Pacific Ocean were investigated and radiation doses to the Japanese and world population from ingestion of seafood contaminated by Fukushima radionuclides were estimated and compared with those from other sources of anthropogenic and natural radionuclides. The total effective dose commitment from ingestion of radionuclides in fish, shellfish and seaweed caught in coastal waters off Fukushima was estimated to be 0.6 ± 0.4 mSv/y. The individual effective dose commitment from consumption of radioactive-contaminated fish caught in the open Pacific Ocean was estimated to be 0.07 ± 0.05 mSv/y. These doses are comparable or much lower than doses delivered from the consumption of natural ^210^Po in fish and in shellfish (0.7 mSv/y). The estimated individual doses have been below the levels when any health damage of the Japanese and world population could be expected.

After the Great East Japan earthquake and subsequent Tohoku tsunami with unexpectedly high waves on 11^th^ March 2011, the Fukushima Dai-ichi Nuclear Power Plant (FDNPP) by force majeure undergone a total damage of the electrical network and of the emergency diesel generators, resulting in the lost of ability to cool three nuclear reactors which were in operation, as well as the fuel storage pools, what resulted in a complex damage of the FDNPP^1^,^2^. Releases of radionuclides during the Fukushima accident were controlled by physical and chemical properties of radioactive elements in the cores of nuclear reactors, mainly by their boiling characteristics. Due to several hydrogen-air explosions, which heavily damaged three nuclear reactors, large amounts of radionuclides were released to the environment^2^,^3^,^4^,^5^,^6^,^7^. Radionuclides were released either in the form of radioactive gases which spread over the world^2^,^8^,^9^, or by direct releases of contaminated fresh water (and later also seawater) to coastal waters^2^,^10^,^11^,^12^. Due to dry and wet depositions of radionuclides from the atmosphere, both terrestrial and marine environment have been contaminated^2^.

Radioactive compounds that accumulate in the food chain, such as radioisotopes of iodine, cesium and strontium dominate in delivering doses to humans and biota. Radiological effects from other radionuclides, e.g. from plutonium isotopes, are usually much smaller than those from the fission products. The release rates of plutonium isotopes during the Fukushima accident were also much smaller than that of radioiodine, radiocesium and radiostrontium^13^,^14^,^15^. The human body absorbs iodine, cesium and strontium readily via inhalation or ingestion, which are then transported by the blood to the human tissues. The ^131^I is rapidly absorbed by the thyroid, and leaves only after its radioactive decay with a half-life of 8 days. Cesium is mainly absorbed by muscles, and due to long physical half-lives of its radioisotopes (30 years for ^137^Cs and 2.1 y for ^134^Cs) it remains in the body until it is excreted (10-100 days biological half-life). The long-lived isotopes of iodine (^129^I, half-life 1.57 × 10^7^ y) and cesium (^135^Cs, half-life 2.95 × 10^6^ y), which are weak beta-emitters, do not contribute significantly to radiation doses. In the case of ^129^I, the levels are useful for estimation of radiation doses due to ^131^I, which because of short time available after the accident usually could not be precisely estimated. The ^129^I has also been frequently used as an isotope trace of movement of oceanic waters^16^. Strontium, on the other hand is absorbed in bones, and because of its long half-life (28 y) it may be radiologically important as well. As there is still on going discussion on effects of low-level radiation doses on humans^17^, their assessments in the case of nuclear reactor accidents have been carried out with the aim to estimate even minimal radiation doses to the public.

There have already been published a few estimations of radiation doses to the public from consumption of seafood contaminated by Fukushima radionuclides^2^,^18^,^19^, however, a complex coverage of the problem using large data sets on activities of radiocesium and radiostrontium in coastal and open ocean seawater and seafood has been missing, as well as a comparison with other sources of radionuclides in the marine environment. The post-Fukushima dose estimations have mostly been carried out for inhalation, external irradiation and consumption of radionuclide contaminated terrestrial food^2^,^20^. The aim of the present paper has been to estimate radiation doses to the Japanese and world population due to the ingestion of seafood contaminated by Fukushima-derived radionuclides (mainly by Cs and Sr radioisotopes), and to compare them with other radionuclide sources in the marine environment (e.g. anthropogenic radionuclides from global fallout, and natural radionuclides). The radiation doses to the public may come from ingestion of seafood collected either in coastal waters and/or in the open ocean.

## Results

### Fukushima radionuclide time series

#### Temporal variations in radionuclide concentrations in surface seawater offshore Fukushima

**Figure 1:**
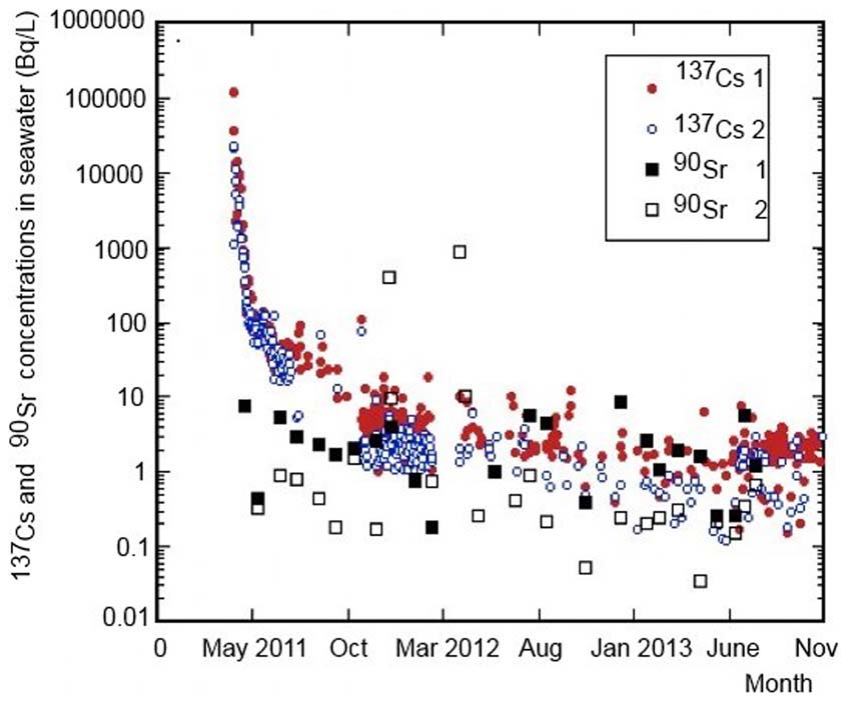
Temporal variations of ^137^Cs and ^90^Sr activity concentrations in surface waters near the north (1) and south (2) water outlets (data from Refs. 4,5,6,7, 10,11,12, 15, 27,28,29). Relative data uncertainties (at 1 sigma) are below 10%.

**Figure 2:**
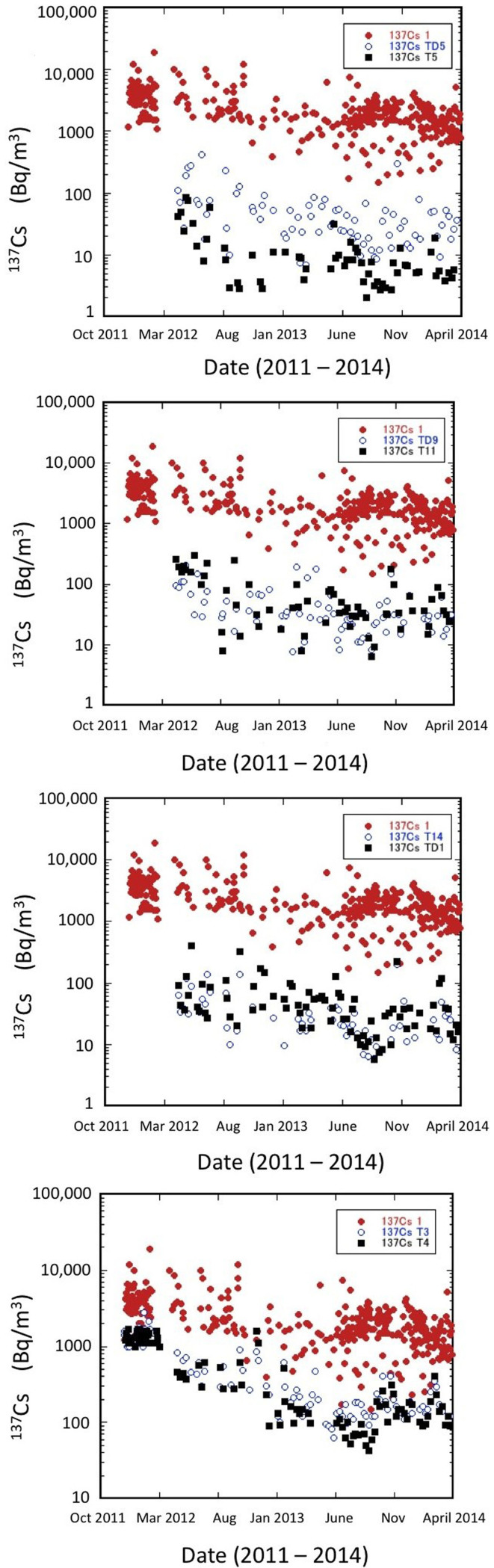
Temporal changes of ^137^Cs activity concentrations in surface waters at monitoring sites within 20 km from the Fukushima Dai-ichi NPP (data from Refs. 5,6,7, 14, 21). Notice remarkable differences between ^137^Cs levels observed close to the FDNPP (St. 1, red dots) and the more distant stations. Relative data uncertainties (at 1 sigma) are below 10%. Positions of monitoring sites are given in Table 1.

We examined temporal variations of ^134^Cs and ^137^Cs activity concentrations in surface waters near the FDNPP which markedly increased due to direct discharges of contaminated water to the sea, as well as due to atmospheric radionuclides depositions^2^,^15^,^16^,^21^,^22^. The ^137^Cs and ^134^Cs levels in seawater near the water outlets from the FDNPP mostly varied during 2012–2014 between 0.1 and 10 kBq/m^3^, however, in March 2011 they reached concentrations up to 100 MBq/m^3^ (Fig. 1). In early April 2011 the ^137^Cs concentrations in surface waters decreased due to ceases of leaks of the contaminated water to the sea and atmospheric emissions/depositions from the FDNPP. During 2011 the ^137^Cs concentration in coastal waters (within 20 km) varied between 0.08 and 8 kBq/m^3^ (Ref. 2). Figure 2 shows the temporal variations of the ^137^Cs concentrations in surface waters at the monitoring sites within about 20 km off Fukushima during the period of November 2011 to March 2014. There have been remarkable differences between the ^137^Cs levels observed close to the FDNPP (St. 1) and more distant stations. The ^137^Cs activity concentrations in surface waters in 2012 were between 0.003 and 2 kBq/m^3^, and during 2013 between 0.002 and 0.6 kBq/m^3^, gradually decreasing with time at all sites.

Due to the influence of the specific current system offshore Fukushima (the Oyashio current bringing cold waters from the north, and the Kuroshio current bringing warm waters from the south), the region is well known for fast transport of seawater to the open Pacific Ocean^23^,^24^. Assuming that the surface ^137^Cs concentrations in seawater within 20 km off the FDNPP decreased exponentially during the period of April 2012 to March 2014, we calculated an apparent half-life of ^137^Cs at each site (Table 1), which ranged from 11 months to 18 months. The longest apparent half-life of ^137^Cs in surface waters appeared at the north and south outlet sites near the FDNPP, whereas the shortest half-lives were observed at the sites distant from the FDNPP. These findings suggest a continuous supply of ^137^Cs into coastal waters near the FDNPP. The spatial pattern of the apparent half-lives is consistent with the previous reports that a source of continuous release of ^137^Cs exists around the north outlet of the FDNPP^22^,^24^.

The ^90^Sr activity concentration in seawater near the same water outlets mostly varied between about 0.1 kBq/m^3^ and about 10 kBq/m^3^, however, during March 2012 its levels raised up to 1 MBq/m^3^ due to sporadic releases/leakages of contaminated waters^5^,^6^,^7^,^14^,^21^,^25^ (Fig. 1). The ^90^Sr/^137^Cs activity ratio in seawater at the same water outlets varied between about 0.005 and 500, indicating large variations in composition of radionuclides in waste waters which were released from the FDNPP to the sea. A comparison of the variable Fukushima ^90^Sr/^137^Cs activity ratios with the global fallout ratio (0.6) indicates large differences in assessing radiation doses from consumption of marine food using both radionuclide release-rates scenarios.

Several incidents with environmental releases of radionuclides from the FDNPP have occasionally occurred during the past three years. As an example, in July 2013, TEPCO announced that underground water including radioactive materials had leaked into the port at the FDNPP^26^. Occasional releases/leakages of contaminated waters could occur from storage tanks and/or stagnant waters situated on the FDNPP site^27^. There is about 500,000 tons of contaminated water stored presently at the site (with about 150,000 tons increase/year), which may represent a serious radiological danger for possible further contamination of seawater offshore the FDNPP^25^.

#### Temporal variations of radionuclides in surface and bottom dwelling fish

**Figure 3:**
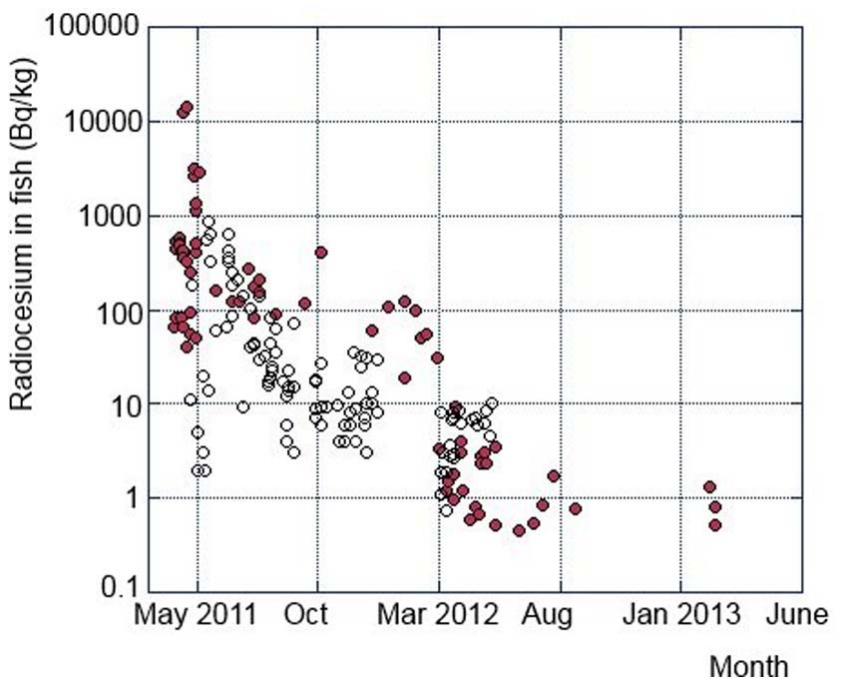
Temporal variations of radiocesium (^134^Cs + ^137^Cs) concentrations in surface-dwelling fish for 2011–2013 (closed circles: Japanese Sandlance, open circles: whitebait); data from Ref. 37. Relative data uncertainties (at 1 sigma) are below 10%.

**Figure 4:**
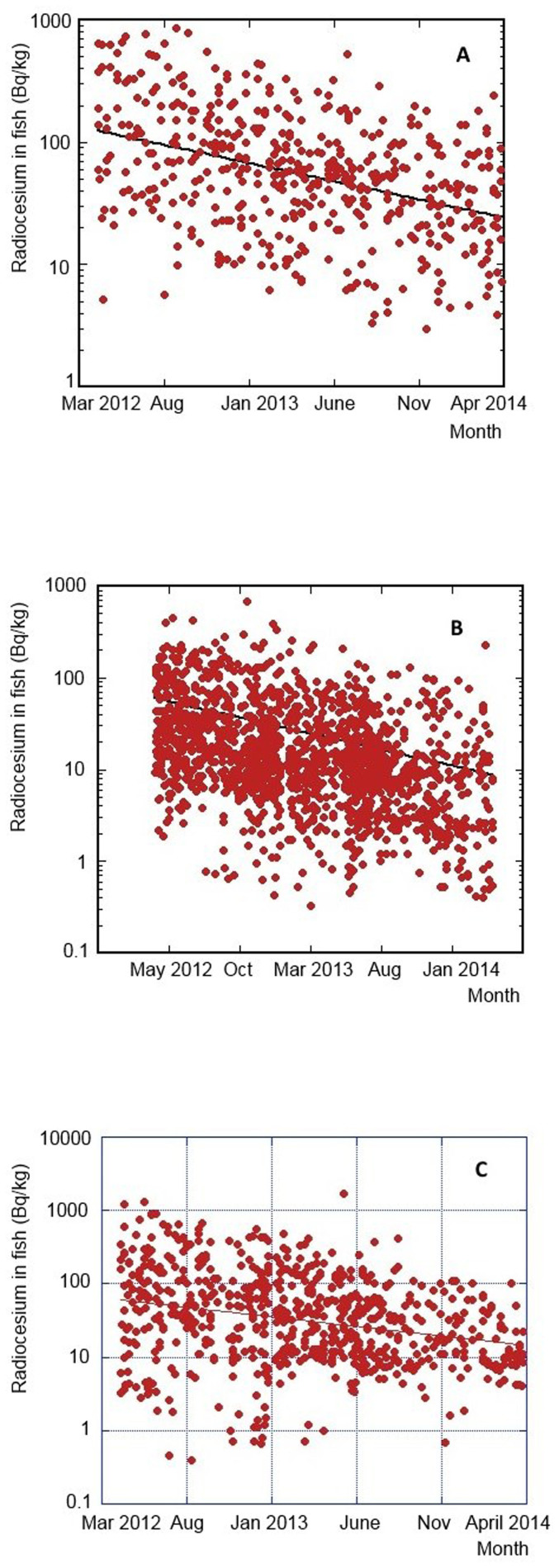
Temporal variations of radiocesium (^134^Cs + ^137^Cs) concentrations in bottom-dwelling fish for 2012–2014 (A: Common Skete, B: Bastard halibut, C: Fat greenling); data from Ref. 36. Relative data uncertainties (at 1 sigma) are below 10%.

After the major deposition of atmospheric radionuclides and the direct releases of contaminated water to the sea, marine biota was extensively contaminated by the FDNPP-derived radionuclides^28^. The Japanese Ministry of Agriculture, Forestry, and Fisheries (MAFF) has been monitoring radionuclides in fish and other seafood products since 23 March 2011^28^,^30^. The ^137^Cs concentrations in fish caught offshore Fukushima during the period of April 2011 to March 2013 varied within several orders of magnitude, from about 0.5 Bq/kg ww (wet weight) to 15 kBq/kg ww for surface-dwelling fish (Fig. 3), and for bottom-dwelling fish they were during the period from April 2012 to April 2014 from about 0.3 Bq/kg ww to 3 kBq/kg ww (Fig. 4). Demersal fish have higher radiocesium levels than other marine fish types, including epipelagic, pelagic and neuston fish, and the radiocesium concentrations in demersal fish showed lower decrease rates than the other marine fish. Major cause of the difference of ^137^Cs decrease rates between surface- and bottom-dwelling fish is due to different living areas. While surface-dwelling fish is migrating wide-sea areas including low contaminated areas, bottom-dwelling fish caught off Fukushima are sedentary^6^. The MAFF results^29^,^30^ revealed that the radiocesium concentrations in epipelagic and neuston fish have been rapidly decreasing with time and most of their radiocesium concentrations were less than detection limit in the mid 2012.

A consumption of seaweeds has also been important part of the Japanese dietary habits. The ^134^Cs + ^137^Cs levels in seaweeds collected offshore Fukushima during the period of May to December 2011 varied from 1000 to 30 Bq/kg (Ref. 2).

We further examined temporal variations of radiocesium concentrations in demersal fish during the period of April 2012 to April 2014 using the MAFF data^30^. The observed ^137^Cs levels were gradually decreasing during the sampling period, although there was large variability of the radiocesium concentrations between fish samples (Fig. 4). Assuming that the radiocesium concentrations showed an exponential decrease with time, the apparent half-lives of radiocesium in Common Skete (*Raja kenojei*), Bastard halibut (*Paralichthys olivaceus*), and Fat greenling (*Hexagrammos otakii*) were calculated to be 10 ± 1, 8.5 ± 0.5 and 12 ± 2 months, respectively, corresponding to 12 ± 1, 10.1 ± 0.6 and 15 ± 3 months of apparent half-lives of ^137^Cs in demersal fish, of a similar time scale as 11–18 months of apparent half-lives of ^137^Cs in coastal waters.

The ^137^Cs concentrations in fish caught in the open ocean before the Fukushima accident were following the decrease of the ^137^Cs concentrations in surface waters of the NW (North-West) Pacific Ocean^33^,^34^. These findings suggest that the radiocesium concentrations in surface waters control trends of the radiocesium concentrations in marine fish, irrespective of species of fish, which lives in the corresponding sea area. For the coastal area off Fukushima, where the water depth is relatively shallow (less than 100 m), no significant difference between ^137^Cs concentrations in surface and bottom waters is expected.

**Figure 5:**
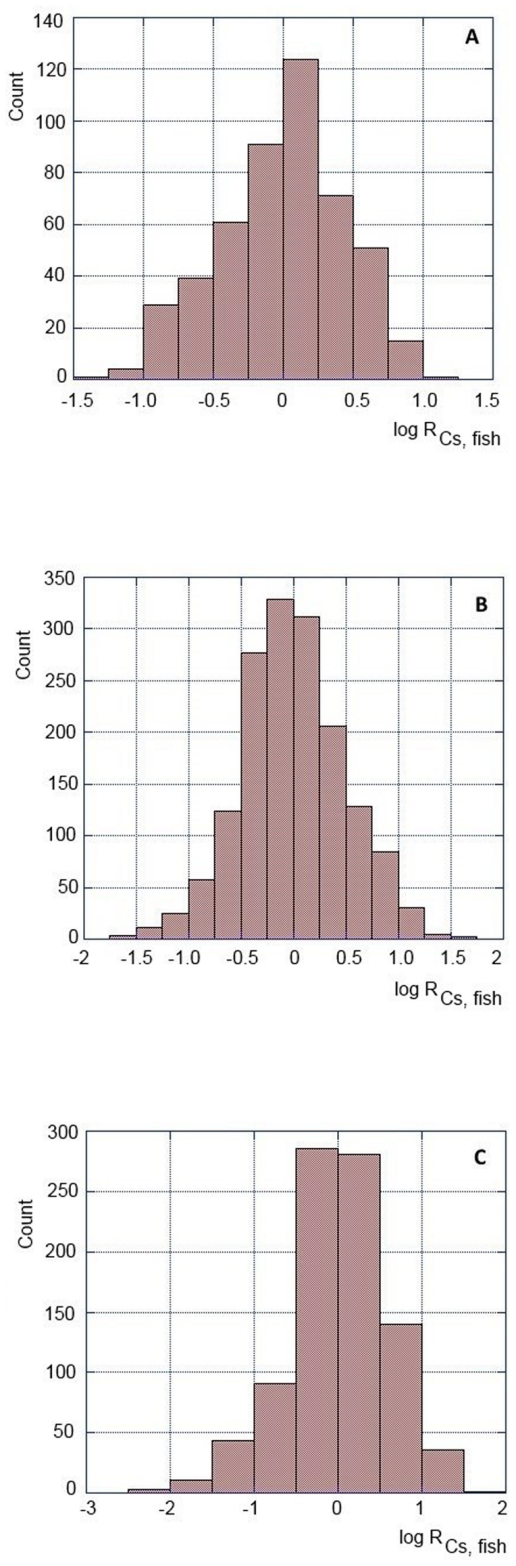
Frequency distributions of logarithmic values of the observed to calculated ratios for bottom-dwelling fish (A: Common Skete, B: Bastard halibut, C: Fat greenling).

The MAFF results revealed that the radiocesium concentrations in some species of demersal fish showed large variability, exceeding the regulatory limit of 100 Bq/kg ww even in early 2014. Therefore Japanese government continues to keep fisheries closed offshore Fukushima. The average values of the radiocesium concentrations in Common Skete, Bastard halibut, and Fat greenling in March 2014, estimated from the best-fit curve (Fig. 4), were 25, 4.7 and 15 Bq/kg ww, respectively. These values were thus significantly lower than the regulatory limit of 100 Bq/kg. To elucidate the amount of scatter in measured values, ratios of measured values to values calculated from the exponential regression (R_Cs,fish_, in which trends of the radiocesium concentrations in fish are reduced), were calculated (Fig. 5). The logarithmic ratios showed normal distribution, in which standard derivations of the logarithmic ratios for Common Skete (number of samples: 487), Bastard halibut (number of samples: 2312), and Fat greenling (number of samples: 688) were 0.449, 0.495 and 0.596, respectively. Taken into account the 95% confidence interval of measured values, most of the observed radiocesium concentrations in Common Skete, Bastard halibut, and Fat greenling were less than 190, 44 and 220 Bq kg^−1^ ww, respectively. The results suggest that the radiocesium concentration in Bastard halibut was within the regulatory limit at the 95% confidence interval on March 2013, whereas the higher measured radiocesium values in Common Skete and Fat greenling, statistically exceeded the regulatory limit in 2014 due to the slow decrease rates of radiocesium in demersal fish.

The large scatter of the measured values of the radiocesium concentrations in demersal fish are primarily attributable to heterogeneous distribution of radiocesium in coastal waters near the FDNPP, which is related to continuous direct releases of contaminated waters, and due to ecological behaviors of demersal fish. The radiation dose due to annual intake of radiocesium in fish, which is important factor to determine the regulatory limit, is statistically related to the mean value of the radiocesium concentrations in fish rather than its maximum value. The large scatter of measured values in fish should be taken into account when to apply the regulatory limit.

For comparison radiocesium levels in other marine biota were in the range 70–430 Bq/kg dw for macroalgae, and 50–400 Bq/kg dw for mussels^34^.

### Radionuclides in seawater and biota of the open Pacific Ocean

^137^Cs and ^134^Cs levels observed in the open NW Pacific Ocean surface waters (east of 143°E) after the Fukushima accident varied during 2011–2013 in the intervals from <1 to 100 Bq/m^3^ (Refs. 2, 21, 35,36,37,38). The levels of these radionuclides observed in fish (Bluefin and Yellowfin tunas and others) caught in the open Pacific Ocean during the period of April 2011 to November 2012, were in the interval from 0.3 to 41 Bq/kg ww^21^,^29^,^30^. Radiocesium concentrations in zooplankton were even lower, <15 Bq/kg dw^39^. The ^90^Sr activity concentrations in the open Pacific Ocean after the Fukushima accident varied between <1 and 10 Bq/m^3^ (Ref. 40). The ^90^Sr levels in fish caught offshore Fukushima were in the interval from 0.01 to 1.2 Bq/kg ww^30^. It is necessary to mention that most of the ^90^Sr concentrations in fish samples (more than 90%) were less than detection limits.

A comparison of natural (^40^K) and anthropogenic (^137^Cs and ^90^Sr) levels in the world ocean and the adjacent seas during pre- and post-Fukushima time is presented in Table 2. It can be seen that the ^137^Cs and ^90^Sr levels in seawater after the Fukushima accident increased at the Fukushima coast by about a factor of 10,000, while the expected maximum increase in the open ocean is due to large dilution in the huge Pacific Ocean only by about a factor of 100 (Refs. 41, 42).

Fortunately we have had at disposal large pre-Fukushima radionuclide data sets stored in the GLOMARD/MARS^41^,^43^ and HAM^44^ databases. Research cruises organized during 1991–2010 helped to establish background (global fallout) radionuclide levels in the NW Pacific Ocean^31^,^32^,^33^, so pre-Fukushima distribution of ^137^Cs, ^90^Sr and other radionuclides in the water column has been well established. Generally, the pre-Fukushima activity concentrations of ^137^Cs and ^90^Sr of global fallout origin in surface waters of the western Pacific Ocean were around 1 Bq/m^3^ for ^137^Cs, and around 0.6 Bq/m^3^ for ^90^Sr (Ref. 44).

### Assessment of radiation doses

The Japanese government has applied very strict regulations for radionuclide content in seafood, decreasing the Japanese limit (sum of ^134^Cs and ^137^Cs) for the Fukushima accident from 500 to 100 Bq/kg ww^45^, so it became by about a factor of four to ten lower than for other Asia and European countries. The Codex value of 1000 Bq/kg ww (recommended by the Codex Alimentarius Commission of the World Health Organization and the Food and Agricultural Organization, http://www.codexalimentarius.org/codex-home/en/), which has been accepted by most of the world countries, is assuring the maximum effective dose limit to population from consumption of seafood of <1 mSv/year. The Japanese approach, which has been thus very conservative, has been based on large consumption amounts of seafood, as well on the fact to make provisional regulation limits to be safer from the point of view of total dose commitments from other radiation/contamination sources, such as inhalation, external irradiation and the ingestion of terrestrial food. National and regional Japanese institutions have carried out extensive monitoring programs to exclude those seafood items, which were over the radionuclide concentration limit. This has also been done outside of Japan, e.g. the monitoring program of the European Union did not find imported seafood, which would be over the claimed Japanese radionuclide limit of 100 Bq/kg ww^46^.

The most suitable way how to calculate the radiation doses from seafood in highly contaminated areas would be to use the maximum permissible radionuclide concentrations in a given type of the seafood. The resulting dose rates can be then adjusted for possible deviations, both in the radionuclide levels in the seafood, as well as in the consumption rate of seafood. The approach based on the regulation limits^1^ is, of course, having a weak point as there could be a hypothetical group of people (e.g. a fisherman family), that will not be covered by a radionuclide screening, and the delivered radiation doses could be thus higher.

#### Radiation doses from consumption of seafood collected in coastal waters

As the radionuclide concentrations in coastal seawater differed in time and space by several orders of magnitude (from about 0.1 kBq/m^3^ to about 100 MBq/m^3^, see Fig. 1) due to different release rate scenarios and with the distance from the shore, to calculate the effective dose commitments is not an easy task. If we take for the average ^137^Cs activity concentrations off Fukushima (20 km radius) during 2011, 2012 and 2013 values of 500 (Ref. 2), 200 and 60 Bq/m^3^ (see Fig. 2), respectively, then the effective dose commitments calculated using the Method 1 from ingestion of seafood contaminated by ^137^Cs and ^134^Cs during 2011, 2012 and 2013 would be 0.3, 0.1 and 0.03 mSv/y, respectively. The total effective dose commitment calculated for the period 2011–2013 is then estimated to be 0.5 ± 0.3 mSv/y (0.4 mSv/y from consumption of fish, 0.03 mSv/y from shellfish and 0.06 mSv/y from seaweed).

Due to rapid changes of radionuclide contents in seawater and in corresponding fish there are also problems with application of the Method 2 for dose assessments. As fish can migrate several tens of kilometers, the radionuclide concentrations in seawater, and resulting radionuclide levels in affected fish could change over the migration distance by several orders of magnitude. This of course should not be a problem for other types of seafood (e.g. seaweed, mussels and shellfish), which are either fixed on the seafloor, or the travelling distances are small.

The majority of radiocesium concentrations measured in fish were in the range 10–1000 Bq/kg ww, although the observed levels varied between 2011–2014 from about 0.5 Bq/kg ww to about 20 kBq/kg ww (Figs. 3 and 4). The average radiocesium levels observed in fish during 2011, 2012 and 2013 were 240, 95 and 40 Bq/kg ww. The corresponding effective dose commitments calculated using the Method 2 from ingestion of seafood contaminated by ^137^Cs and ^134^Cs during 2011, 2012 and 2013 would be 0.16, 0.07 and 0.03 mSv/y, respectively. The total effective dose commitment calculated for the period 2011–2013 is then estimated to be 0.3 ± 0.2 mSv/y, in a reasonable agreement with the value of 0.5 ± 0.3 mSv/y calculated using the Method 1. The results presented in Fig. 5 suggest, however, that the higher measured radiocesium values in Common Skete and Fat greenling statistically exceeded the regulatory limit even in 2014. Therefore to be on a conservative side, for calculation of dose for the year 2013 we also use the screening value of 100 Bq/kg ww, which would result then in more robust dose estimation as the available fish data have been too scarce. The resulting total effective dose commitment for the period 2011–2013 will be then 0.4 ± 0.3 mSv/y (0.3 mSv/y from consumption of fish, 0.03 mSv/y from shellfish and 0.03 mSv/y from seaweed). By combining results obtained by both methods we may conclude that the radiation doses from consumption of ^137^Cs and ^134^Cs in contaminated seafood collected in coastal waters during 2011–2013 should be 0.5 ± 0.3 mSv/y.

For a critical group consuming fish with ^137^Cs content of 1000 Bq/kg ww, and the seafood amounts by a factor of 4 higher as the Japanese average per year (i.e. the total consumption of seafood of 100 kg/y), the total dose including ^134^Cs and other pathways will be about 3 ± 2 mSv/y, slightly higher than the world average dose from natural radiation sources (2.4 mSv/y).

#### Radiation doses from consumption of seafood from the open Pacific Ocean

There has been a fast seawater transport due to the Kuroshio Current and the Kuroshio Extension from the Japanese coast to the open North Pacific Ocean^23^,^24^. Therefore in such situation, and for some fish species, which migrate in the ocean for large distances, it is difficult to estimate radiation doses for public from fish caught in the Pacific Ocean. For example some types of fish (e.g. Yellow tuna or Bluefin tuna) can migrate from Japan to California coastal waters^18^. Therefore the dose assessment was done using the Method 1 by multiplying radionuclide concentrations in water with the concentration factors *(CF)_j,k_*.

As the radionuclide data density for the open ocean is very sparse, we shall apply a very conservative approach and take for the ^137^Cs activity concentration in the NW Pacific Ocean the maximum observed value of 100 Bq/m^3^ (Refs. 2, 36, 47). The effective dose commitment for ingestion of ^134^Cs and ^137^Cs in fish caught in 2011–2013 at the open NW Pacific Ocean was estimated to be 0.06 ± 0.04 mSv/y for all pathways. For more realistic average ^137^Cs activity concentration in the NW Pacific Ocean of about 10 Bq/m^3^ (Ref. 2), the calculated dose would be only 0.006 mSv/y. If we calculate the dose following the Method 2 using the maximum measured ^137^Cs activity in fish in the open NW Pacific Ocean (40 Bq/kg ww^30^), we get a value of 0.03 ± 0.02 mSv/y. The estimated dose is much lower than the annual dose limit for public from external sources (1 mSv/y) recommended by ICRP and IAEA. Because the dose is proportional to the consumption amount of contaminated seafood, most public in the world will get even lower doses than the Japanese population.

#### Radiation doses from other radionuclides

Atmospheric and liquid releases of other radionuclides from the damaged FDNPP were much lower when compared with cesium radioisotopes. From about 30 radionuclides released from the FDNPP and deposited or directly released to the marine environment^48^, their effects on delivering internal radiation doses due to the ingestion of contaminated seafood have been much smaller than in the case of cesium radioisotopes^23^. Only ^90^Sr because of its radiological significance and large release rates may be considered important for delivering radiation doses to the public from consumption of contaminated seafood^2^. The available data on ^90^Sr activity concentrations in seawater and biota are, however, very limited when compared with radiocesium data. As the ^90^Sr/^137^Cs activity ratios in Fukushima coastal waters varied considerably after the Fukushima accident (from 0.005 to about 500) because of different release rates of both radionuclides, it is difficult to assess effective dose commitments due to ^90^Sr from consumption of seafood. A maximum effective dose due to ^90^Sr from ingestion of seafood, however, may be calculated using the average ^90^Sr level of 1 kBq/m^3^ (Fig. 1) observed near the Fukushima coast, the dose conversion factor for ^90^Sr of 2.8 × 10^−8^ Sv/Bq^49^, and concentration factors listed in Table 3. A maximum effective dose commitment for ingestion of ^90^Sr in fish caught in 2011–2013 at the Fukushima coast near the FDNPP was calculated to be 0.05 mSv/y. Taken into account the results that the ^90^Sr levels in the open NW Pacific waters were less than 10 Bq/m^3^ (Ref. 42), the effective dose commitment for ingestion of ^90^Sr in seafood collected in the NW Pacific Ocean should be less than 0.5 μSv/y. If we use for the dose calculation the maximum observed ^90^Sr activity in fish caught in the NW Pacific Ocean (1 Bq/kg ww^32^), we get a value of 0.7 μSv/y. The ^90^Sr contribution to the total delivered doses may be thus about 10% for consumption of both the coastal and open ocean fish. The contribution of other radionuclides to the total delivered dose may be only 1% (Ref. 23).

#### Dose uncertainties

There are several factors, which could contribute to the dose uncertainties. In the Method 1 the dominant contribution is from the estimation of a proper radionuclide concentration in seawater. As we took a rather conservative approach, the ^137^Cs radionuclide concentrations in coastal waters used in calculations were at the upper side of observed values. Larger variations in radionuclide levels are expected for open ocean radionuclide concentrations^23^, where we used either the maximum observed ^137^Cs value of 100 Bq/m^3^, or an expected average value of 10 Bq/m^3^. According to IAEA^50^ the uncertainties in dose coefficients and concentration factors for cesium in fish and shelf fish are estimated at 10%. Marine food intake rates of radionuclides are estimated to be within 20% (Ref. 51). In the Method 2 the dominant contribution to the dose uncertainty may come from the estimation of a radionuclide concentration in seafood. As we did not use in the dose calculations ^137^Cs levels below the screening value (100 Bq/kg ww), this approach should be conservative for a general public as seafood with higher radionuclide levels should not be available on the market. The other parameters used in the Method 2 have similar uncertainties as in the Method 1.

Our approach has been conservative also in the estimation of seafood consumption, as we expected that the marine products are consumed as complete samples. This is correct, e.g. in the case of shellfish, however, in the case of fish only about 50% is really consumed. Also we do not take into account losses due to cooking (e.g. a transfer of radionuclides from meat to non-eatable liquid or oil), which can represent up to about 70% of the total marine product^52^. All these factors contributed to the final estimation of uncertainties. If we assume that all the mentioned uncertainties are independent of each other, the total of the estimated uncertainties in the calculations can be worked out to be ~60% for estimates of ^137^Cs doses due to the consumption of fish and shellfish using Method 1 (water data) and ~70%, using Method 2 (biota data). The estimated uncertainties are comparable with other dose assessment exercises^50^,^51^. Our approach has been, however, very conservative, therefore the estimated doses can be considered as the maximum doses delivered to the Japanese and world population from consumption of seafood.

## Discussion

The dominant radiation doses to the public from nuclear reactor accidents are usually from inhalation and from external irradiation from radioactive clouds and from radionuclides deposited on the ground. With increasing distance from the FDNPP, the doses will decrease, and later the doses from ingestion of contaminated food will dominate, however, they will be usually lower than doses from inhalation^50^. The doses received from ^137^Cs via marine foods are much lower than those received from terrestrial foods. If the terrestrial and the marine environments received the same deposition of ^137^Cs per unit area, the dose commitment received by man from the seafood will typically be 2 orders of magnitude less than that received from the terrestrial food-chain^50^,^53^. This is also supported with the data obtained for the Fukushima case when in the two most affected hot spots in the Fukushima Prefecture (Iitate village and Namie town) the estimated radiation effective doses for the first year ranged from 12 to 25 mSv^54^. On the other hand, the doses from ingestion of seafood were estimated to be below 1 mSv/y. The radiation doses in prefectures around the FDNPP were well below the deterministic levels, and therefore health effects are not expected to occur in the general population. The impact of the Fukushima accident was also kept well below the 50 mSv/y limit for the statistical risk of cancer^54^,^55^. In some areas around the Fukushima NPP, however, the intervention level of 10 mSv was reached which required governmental action on the evacuation and food control, as it has been done. Generally, higher individual doses could be due to medical radiodiagnostics, ranging from 0.01 mSv/y for a dental X-ray test up to 30 mSv/y for CT and PET scans or similar nuclear medicine diagnostics. More information on the Fukushima-derived terrestrial radiation doses may be found in the report^54^, recently published by the World Health Organization.

The impact of the Fukushima accident on the total environment (citizens, fauna and flora) has been much smaller when compared with the Chernobyl accident, although both accidents were classified equally as no. 7 on the INES scale^56^. The Chernobyl accident heavily affected mainly the people working in the Chernobyl area during emergency actions, plus evacuees from the contaminated zone, and residents of control zone and other contaminated zones in Ukraine, Belarus and Russia^57^. Evacuees from the contaminated zone got an average dose in 1986 of 30 mSv. Residents of other contaminated areas got during 1986–2005 an average accumulated dose of 10–20 mSv. The individual doses to European population outside of the former Soviet Union were generally below the limit of 1 mSv/y^57^.

The global collective dose commitments from ^137^Cs in seafood contaminated due to the Chernobyl accident has been estimated to be 2,000 man Sv^50^. On the other hand, authorized liquid radioactive discharges from the nuclear reprocessing facilities in Sellafield (UK) and La Hague (France) contributed about 4,000 man Sv^50^. The total collective dose commitment from marine-derived ^137^Cs from global fallout, liquid radioactive discharges in Europe, and the Chernobyl accident is 14,000· man Sv, which corresponds to half of the dose received in one year from ^210^Po (natural alpha-emitter in the ^238^U decay chain) consumption in seafood^50^.

The collective effective dose commitments estimated in the MARDOS project^50^,^51^,^58^ for the consumption of seafood collected in FAO fishing areas^59^ of the world ocean in 2000 was mainly due to ^210^Po, even in such areas as the European seas (3,300 man Sv for ^210^Po vs. 56 man Sv for ^137^Cs), which were affected by radioactive discharges from the nuclear reprocessing facilities in Sellafield and La Hague^50^. In the NW Pacific fishing area the ^210^Po dominates again over the ^137^Cs (16,300 man Sv for ^210^Po vs. 18 man Sv for ^137^Cs), confirming that the consumption of seafood in this part of the world is much higher than in other fishing areas, as the ^210^Po activity concentration in seawater (around 1 Bq/m^3^) is uniform over the world ocean^50^. The collective effective dose commitment from fish and shellfish caught in 2000 for global population was estimated to be 100 man Sv for ^137^Cs in fish and 7 man Sv in shellfish, 10,000 man Sv for ^210^Po in fish and 20,000 man Sv in shellfish. The contribution of ^137^Cs to the collective effective dose commitment from fish and shellfish consumption was thus negligible, below 1% of that for ^210^Po.

The estimated radiation doses to the public from consumption of contaminated seafood presented in this paper may be compared with only a few previous marine studies as mostly terrestrial dose assessments were carried out^2^,^20^. Pacific bluefin tuna caught in the open ocean would result in radiation doses to US population due to ingestion of ^137^Cs of 1–5 μSv/y^18^,^19^, what is in agreement with our estimation of 6 μSv/y from fish caught in the NW Pacific Ocean. A detail marine dose assessment from consumption of ^137^Cs and ^134^Cs in seafood collected in coastal waters of Japan (discussed for 2011 and 2012 using much smaller radionuclide data sets) resulted with total dose of 0.6 mSv/y (Ref. 2), what is in agreement with the present value of 0.4 ± 0.2 mSv/y estimated for the same time period 2011–2012.

Individual dose commitment from consumption of radiocesium and radiostrontium in seafood collected in Japan coastal waters of the Pacific Ocean in 2011–2013 was 0.6 ± 0.4 mSv/y. Although this dose is by about four orders of magnitude higher than the pre-Fukushima dose from global fallout (0.05 μSv/y calculated for the ^137^Cs and ^90^Sr contents in seawater of 1 Bq/m^3^), it is below the maximum permissible annual dose to the public from external sources (1 mSv/y), or the world average dose from natural sources (2.4 mSv/y). The estimated dose is comparable to the annual dose due to the ingestion of ^210^Po in fish and shellfish (0.7 ± 0.4 mSv/y).

Individual dose commitment from consumption of radiocesium and radiostrontium in fish caught in the open NW Pacific Ocean in 2012–2013 is 0.07 ± 0.05 mSv/y, what is about three orders of magnitude above the pre-Fukushima dose. This dose is comparable to the dose due to the consumption of natural ^210^Po in fish, and by 10-times lower than the dose due to the consumption of natural ^210^Po in shellfish.

## Methods

For assessment of radiation doses we shall follow the International Atomic Energy Agency's (IAEA) project on Marine Radioactivity Dose Assessment (MARDOS)^50^,^51^, in which the radiation doses from consumption of marine food were calculated by two different methods.

### Method 1

This method uses the estimated activity concentrations of ^137^Cs in seawater, and recommended concentration factors. The effective dose commitment (S) from consumption of seafood is then calculated using the formula^2^
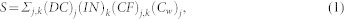
where the *(DC)_j_* represents the dose coefficient for a radionuclide *j* (Sv/Bq), the *(IN)_k_* represents the averaged intake rate of a marine product *k* (kg/y), the *(CF)_j,k_* represents the concentration factor for a radionuclide *j* and a product *k*, and the *(C_w_)_j_* represents the concentration of a radionuclide *j* in seawater (Bq/kg). The dose coefficients, *(DC)_j_*, for a nuclide *j* were obtained from the ICRP (International Commission on Radiological Protection) report^47^. In the case of ^137^Cs and ^134^Cs the values of 1.3 × 10^−8^ Sv/Bq and 1.9 × 10^−8^ Sv/Bq were used, respectively. The averaged intake rate, *(IN)_k_*, of a marine product *k* by Japanese public was estimated from the statistical record of the Ministry of Health, Labor and Welfare (MHLW)^60^. Table 3 also lists the average intake rates *(IN)_k_* used in the calculations, estimated for the Japanese population. The IAEA recommended concentration factors^61^, *(CF)_j,k_*, were used in these calculations (Table 3).

### Method 2

This method uses the estimated radionuclide concentrations in seafood and dose conversion factors. The effective dose commitment from consumption of seafood is then calculated using the formula^2^
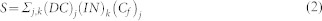
where the *(DC)_j_* is the dose conversion factor for a radionuclide *j* (Sv/Bq), the *(IN)_k_* is the averaged intake rate of a marine product *k* (kg/y), and the (C_f_)_j_ is the concentration of a radionuclide *j* in seafood (Bq/kg). The concentration factors and average intake rates of marine products used in these calculations are listed in Table 3.

## Author Contributions

P.P.P. wrote the main manuscript text and calculated doses. K.H. wrote the text about radionuclide variations and prepared figures. Both authors reviewed the manuscript.

## Figures and Tables

**Table 1:** Apparent half lives of ^137^Cs concentrations in coastal waters near the FDNPP during the period from April 2012 to March 2014

Site name	Location	Half life (month)
T14	37° 32.85′ 141° 04.26′	14 ± 2
TD1	37° 30.02′ 141° 04.33′	13 ± 2
T1	37° 25.63′ 141° 02.58′	18 ± 3
T2	37° 25.14′ 141° 02.56′	18 ± 4
T3	37° 19.44′ 141° 01.61′	11 ± 2
T4	37° 14.47′ 141° 02.56′	11 ± 3
TD5	37° 24.99′ 141° 04.32′	12 ± 2
T5	37° 24.99′ 141° 11.17′	11 ± 2
TD9	37° 20.01′ 141° 04.33′	15 ± 3
T11	37° 14.47′ 141° 04.12′	12 ± 3

**Table 2:** Comparison of natural (^40^K) and anthropogenic (^137^Cs and ^90^Sr) levels in surface seawater in the world ocean and the adjacent seas during pre-Fukushima (adjusted to 2010) and post-Fukushima time

Sea	^40^K (Bq/m^3^)	^137^Cs (Bq/m^3^)	^90^Sr (Bq/m^3^)	References
World ocean	12,000	1–2^a^	0.6–1.2^a^	35,41,58
Baltic Sea	11,500	35^b^	6^b^	58
Irish Sea	11,500	48^c^	40^c^	58
North Sea	11,500	4^c^	3^c^	58
Open Pacific Ocean	12,000	1–100^d^	1–10^d^	2,42
Offshore Fukushima	11,500	10^2^–10^8^ d	10^2^–10^6^ d	4,5,6,7,10,11,12,15,21
Concentrations used in dose calculations:				
Coastal waters		2000^d^	1000^d^	4,5,6,7,15,29,30
Open NW Pacific		100^d^	10^d^	2,36,37,38,39,40,42

^a^Global fallout.

^b^Chernobyl impact.

^c^Impact of the Sellafield and La Hague reprocessing facilities.

^d^Fukushima impact.

**Table 3:** Diet habits and concentration factors

Seafood	Food intake^60^ (g/day)	Concentration Factor^61^ ^134^Cs	Concentration Factor^61^ ^137^Cs	Concentration Factor^61^ ^90^Sr
Fish	64	100	100	3
Crustaceans	5.4	50	50	5
Shellfish	3.5	60	60	10
Seaweed	10	50	50	10
